# First case report of *Schistosoma japonicum* in Nepal

**DOI:** 10.1099/acmi.0.000117

**Published:** 2020-03-27

**Authors:** Dipendra Bajracharya, Sanjeet Pandit, Durga Bhandari

**Affiliations:** ^1^​ Medical Laboratory Technologist, CIWEC Hospital and Travel Medicine center, Kathmandu, Nepal; ^2^​ Clinical Microbiologist, National Public Health Laboratory, Kathmandu, Nepal; ^3^​ M.D. Internal Medicine, CIWEC Hospital and Travel Medicine center, Kathmandu, Nepal

**Keywords:** *Schistosoma japonicum*, Schistosomiasis, first case report, CIWEC Hospital, Nepal

## Abstract

**Background:**

Schistosomiasis, globally, is significant public as well as veterinary health problem as it is associated with a wide range of clinical conditions in humans and animals. Schistosomiasis is mostly caused by the following species of genus *Schistosoma*: *Schistosoma japonicum*, *Schistosoma haematobium, Schistosoma mekongi, Schistosoma intercalatum Schistosoma guineensis, Schistosoma malayensis* and *Schistosoma mansoni. S. japonicum* might be considered as the most pathogenic among these species as the clinical disease caused by this parasite ranges from mild diarrhea, nausea, Katayama fever, portal hypertension, splenomegaly and ascites to liver cirrhosis and fibrosis. *S. japonicum* has been commonly encountered in China, the Philippines and Indonesia. According to WHO, at least 220.8 million people required preventive treatment for schistosomiasis in 2017 but only 102.3 million people were reported to have been treated. To our knowledge, there are no cases reported from Nepal. Hence, this is the first reported case of *S. japonicum* in Nepal.

**Case presentation:**

A case of acute schistosomiasis due to *S. japonicum* was identified in CIWEC Hospital and Travel Medicine Center, Kathmandu, Nepal. The patient arrived with gastrointestinal symptoms without any pre-existing chronic illness with no evidence of travel outside of Spain since last August, but had travelled to many other countries 2 years ago. Timely diagnosis by stool routine and microscopic examination and formal-ether concentration technique led to successful treatment of the disease.

**Conclusion:**

As the parasite has not been reported to date in Nepal, many people are unaware of its mode of infection and pathogenesis. Many laboratory workers are heedless with the egg of the parasite due to which this parasite might be misdiagnosed or undiagnosed. This case report might help laboratory workers to be sentient about the parasite and further diagnosis in future.

## Background

Genus *Schistosoma* (blood fluke) belongs to phylum Platyhelminthes, class Trematoda, subclass Digenea and family *Schistosomatidae*. Schistosomiasis is one of the oldest known diseases in human history caused due to schistosomes. Many species of genus *Schistosoma* have been discovered from long ago. *Schistosoma japonicum,* causing Katayama disease, was first recognized by Akira Fujinami in 1847. Fujinami and Nakamura were the first to report skin infection by *S. japonicum* cercariae in different mammals in 1909 [1]. The worm was discovered by Fujiro Katsurada in 1904 [2]. It was described in the snail host by Miyairi and Suzuki in 1913 and later the life cycle was described by Leiper *et al*. [3, 4].

*Schistosoma* species are dioecious trematodes. The adult schistosomes live in pairs and are approximately 1 to 2 cm long [4]. The eggs of schistosomes are the diagnostic base for the laboratory and species identification. *S. japonicum* might be considered as the most pathogenic species among all species of schistosomes being a causative agent for a wide range of complications like Katayama fever, liver cirrhosis, pulmonary fibrosis, portal hypertension, splenomegaly and neuroschistosomiasis [4].

Schistosomiasis is generally prevalent in tropical and subtropical regions, especially in poor communities where there is no proper access to safe drinking water and adequate sanitation. It is estimated by WHO, that at least 90 % of those requiring treatment for schistosomiasis live in Africa. *S. japonicum* has been commonly encountered in China, the Philippines, Indonesia, etc [3, 5, 6]; to date, we are not aware of any documented case acquired in Nepal. Therefore, we report the first case of Schistosomiasis caused by *S. japonicum* at CIWEC Hospital and Travel Medicine Center, Kathmandu, Nepal.

The CIWEC hospital is a specialized clinic for travellers situated in Kathmandu and is a geo sentinel surveillance site. CIWEC Hospital sees around 8000 patients per year, predominantly travellers. Approximately 30 % of the case load consists of diarrheal disease; altitude-related illness and febrile illness make up significant parts of the caseload.

A written informed consent has been taken from the patient for necessary investigations and case publication in scientific journals.

## Case presentation

A 23-year-old female presented at CIWEC hospital as an out-patient on 8 August 2018, with clinical symptoms of passage of loose watery stools, four–five episodes per day from the previous 2 days with no blood or mucus in the stool as per macroscopic observation. It was associated with fever one episode one day prior to arrival to hospital, which resolved on its own. She had one episode of vomiting on the day of arrival to the hospital. She felt bloated and had a lot of gurgling sounds in her abdomen. Her appetite was normal and she had no nausea. She had no history of any chronic or pre-existing illness.

Regarding her travel history, she had not visited different countries in Europe since 2 years ago. There was no history of travel outside of Spain since last August. She is a non-vegetarian, which would include eating fish on and off in her diet. She had arrived in Nepal in July and had been working as a volunteer in two orphanages in Kathmandu. She admitted that she was not very punctual with her hand hygiene as she would often play with the soil and mud in the orphanage.

Physical examination showed blood pressure 110/60 mmHg, temperature 38.3 ^o^ C, and pulse rate 111 per minute, respiration rate 18 per minute. The patient was given paracetamol orally for fever management.

After physical examination, blood and stool samples were collected for laboratory investigations.

### Haematological investigation

The haematological investigations showed that blood cell parameters and indices were in the acceptable range (Table 1).

**Table 1.** 

Complete blood count	Results
Haemoglobin (12–16 g dl^−1^)	12.7 g dl^−1^
Total red cell count (4.2–5.2×10^6^ µl^−1^)	4.27×10^6^ µl^−1^
Hematocrit (37–48 %)	37.5 %
MCV (80–100 fl)	87.8 fl
MCH (27–32 pg)	29.7 pg
MCHC (32–36 %)	33.9%
Total white cell count (4–10×10 ^3^ µl^−1^)	5.3×10 ^3^ µl^−1^
**Differential Leucocyte count**	
Neutrophils (40–70 %)	55
Lymphocyte (20–45 %)	43
Monocyte (2–10 %)	02
Eosinophil (0–6 %)	00
Basophil (0–2 %)	00
Platelet count (150–450×10^3^ µl^−1^)	248×10^3^ µl^−1^

- **Biochemical findings**

All the biochemical parameters, i.e. blood glucose, BUN, creatinine, electrolytes, liver enzymes and pancreatic enzymes were also found normal. (Table 2)

**Table 2.** 

Tests (reference range)	Results/findings
Glucose, random (70–140 mg dl^−1^)	85 mg dl^−1^
**Renal function tests**	
Blood Urea Nitrogen (7–21 mg dl^−1^)	7.1 mg dl^−1^
Serum creatinine (0.6–1.4 mg dl^−1^)	0.8 mg dl^−1^
Sodium (135–146 mEq l^−1^)	139 mEq l^−1^
Potassium (3.5–5.2 mEq l^−1^)	4.2 mEq l^−1^
**Liver function tests**	
Total bilirubin (<1.4 mg dl^−1^)	0.3 mg dl^−1^
SGOT (AST) (<40 IU l^−1^)	15 IU l^−1^
SGPT (ALT) (<40 IU l^−1^)	16 IU l^−1^
ALP (90–460 IU l^−1^)	113 IU l^−1^
Total Protein (6–8 gm dl^−1^)	6.5 gm d^−1^
Albumin (3.3–5.5 gm dl^−1^)	3.5 gm dl^−1^
**Pancreatic function test**	
Serum amylase (25–90 IU l^−1^)	29 IU l^−1^

- **C-reactive protein**: Negative (less than 5 mg l^−1^)
- **Stool examination**

Stool examination was performed by direct wet mount method and formal ether concentration method. Stool was collected from the patient and direct wet mount and formal ether concentration techniques were performed.

Macroscopically, the stool colour was brown and was soft in consistency without any evidence of visible blood.

**Fig. 1.**
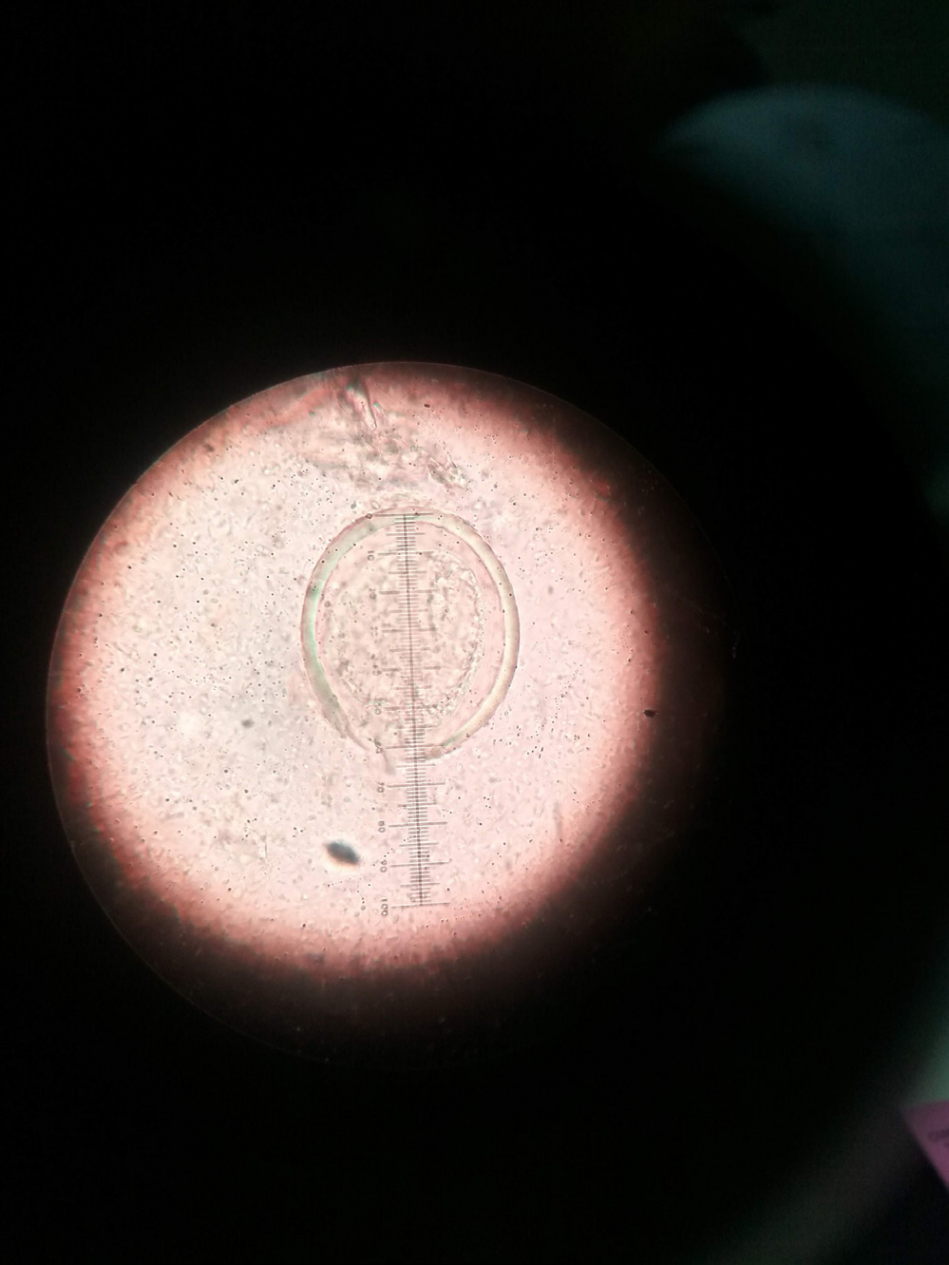
Width of *S. japonicum* egg (x100).

**Fig. 2.**
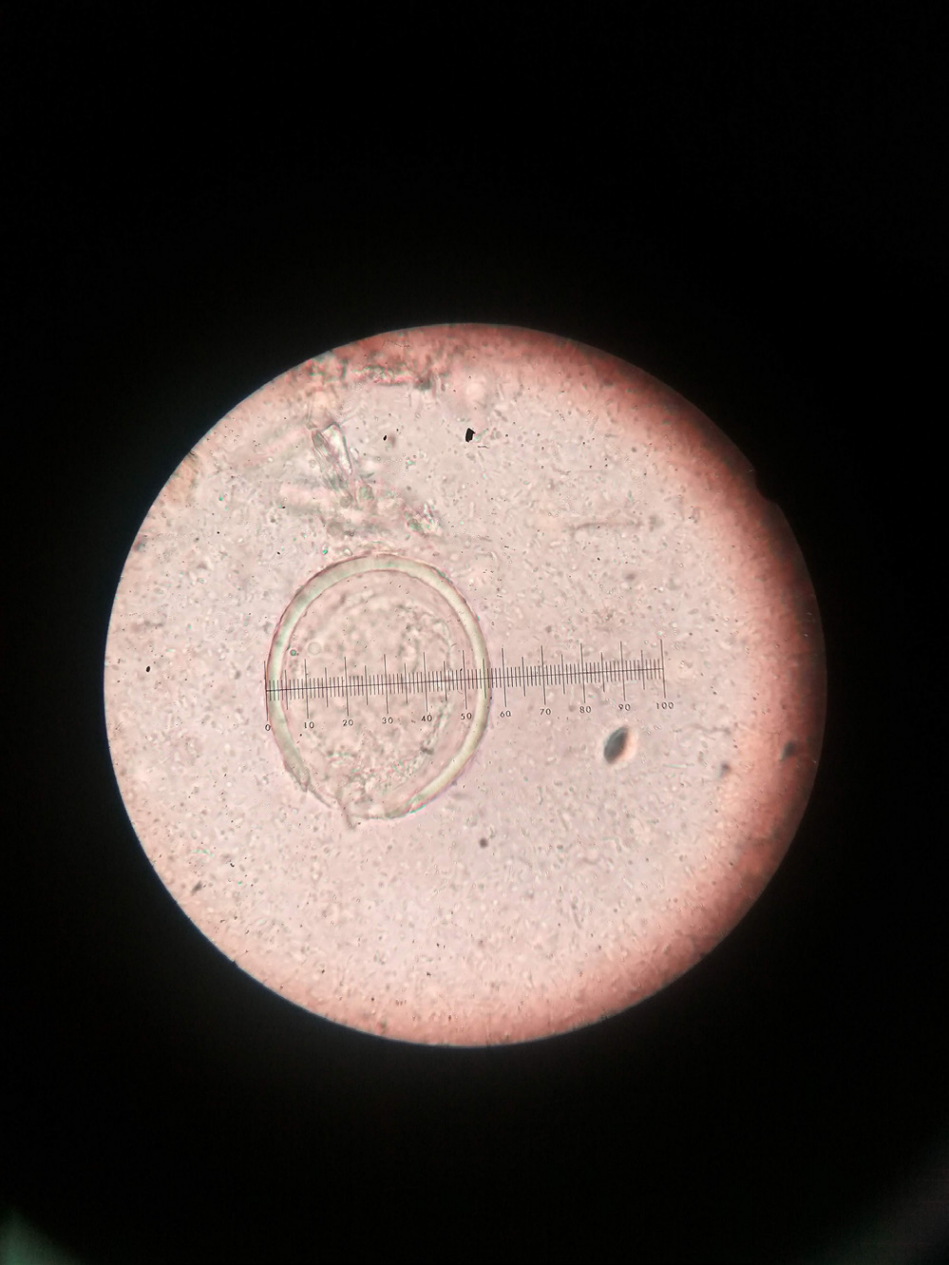
Length of *S. japonicum* egg (x100).

**Fig. 3.**
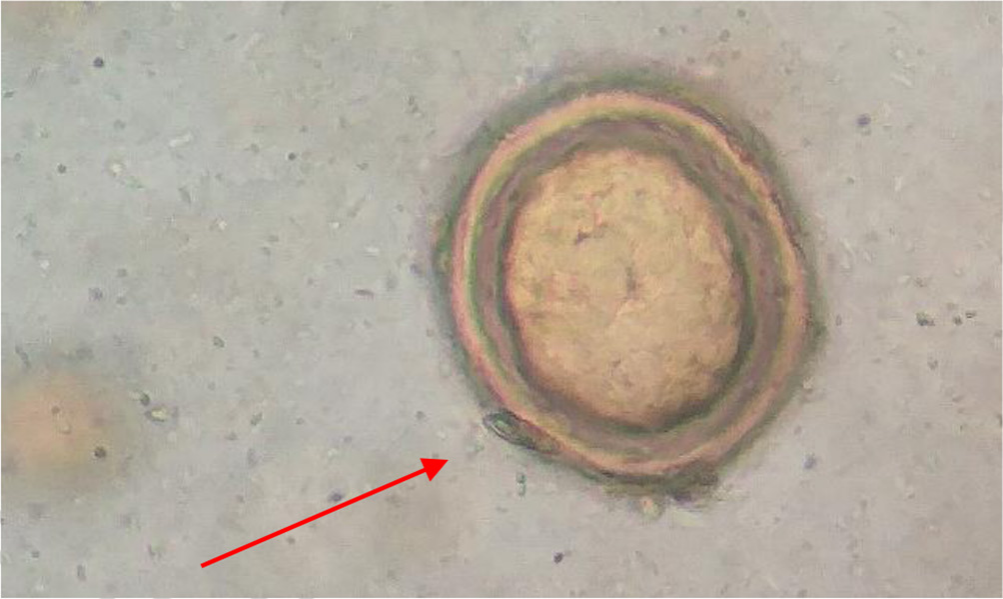
*S. japonicum* egg where the red arrow indicates a knob (x40).

**Fig. 4.**
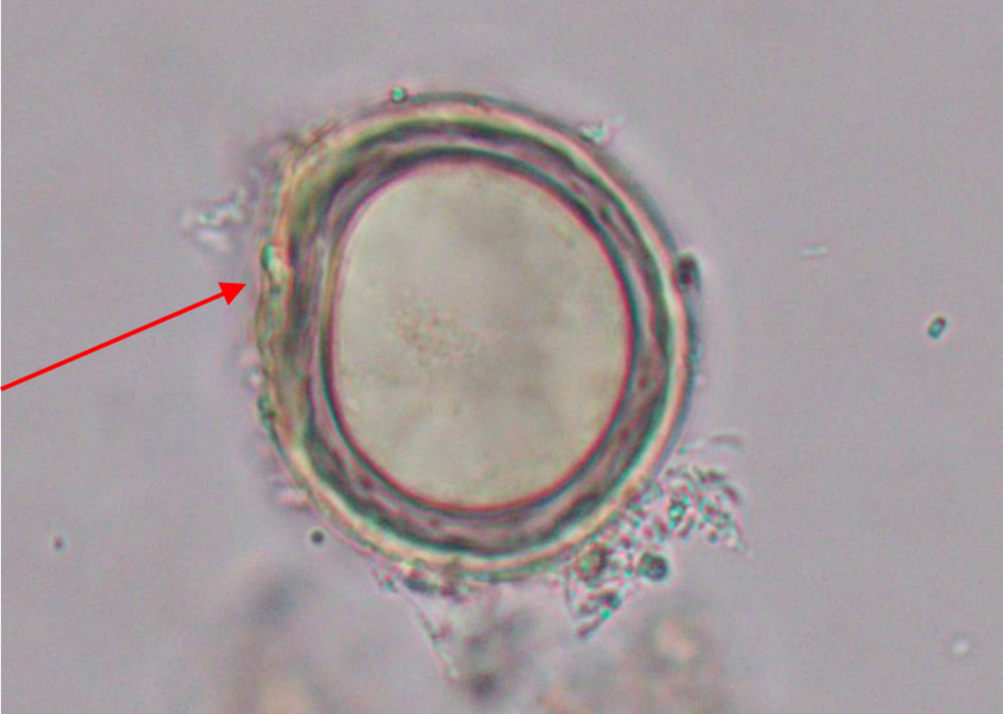
*S. japonicum* egg where the red arrow indicates a knob (x40).

**Fig. 5.**
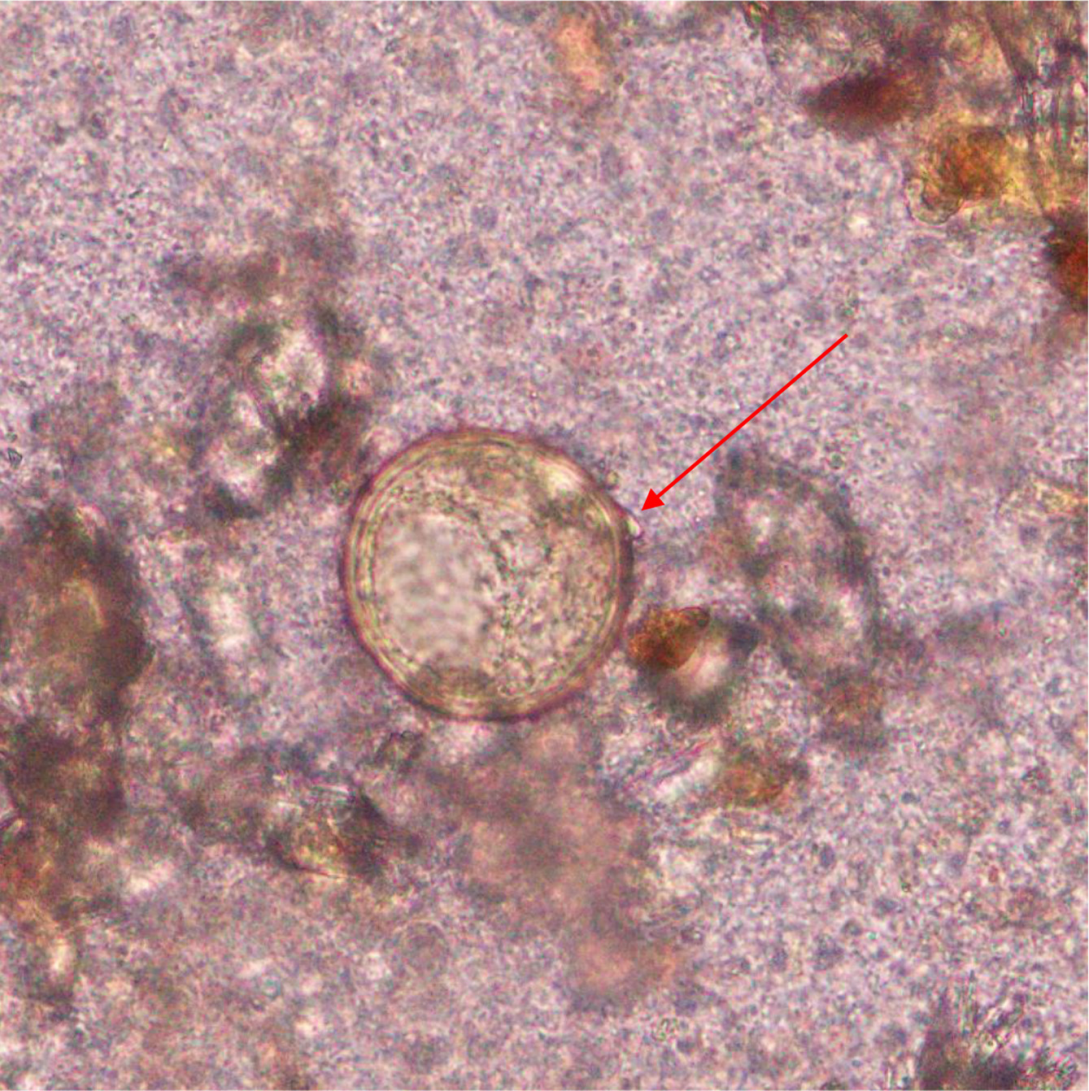
*S. japonicum* egg where the red arrow indicates a knob (x100).

**Fig. 6.**
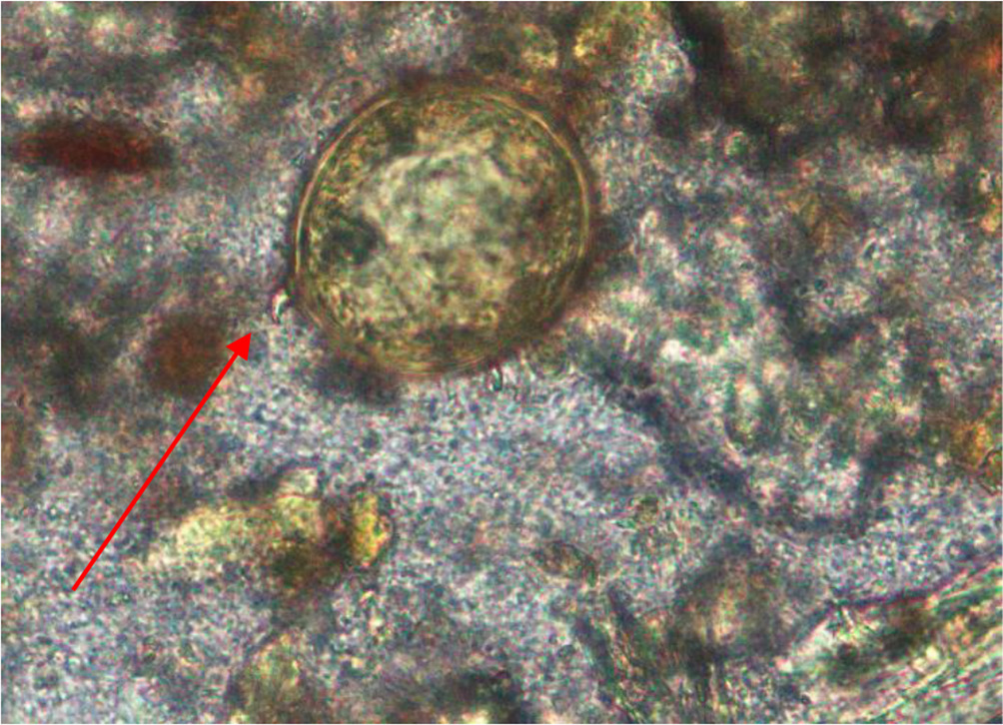
*S. japonicum* eggwhere the red arrow indicates a knob (x100).

On microscopic examination, pus cells, mucus and red blood cells were not seen in direct wet mount of stool. However, there were plenty of eggs of *S. japonicum* (Figs 1–6) observed, which was spotted by Medical Technologist of CIWEC Hospital, Lainchaur and confirmed by the Clinical Microbiologist of National Public Health Laboratory (Reference Laboratory), Government of Nepal, Teku.

The egg of the parasite was clear and pale yellow colour with a knob at one end (indicated by the red arrow in the images) (Figs 3–6). The eggs measured approximately 60–65 µm in length and 50–55 µm in breadth (Figs 1 and 2).

### Follow up

#### Physical examination

The patient had no complaint of fever, headaches and abdominal bloating. Her temperature, pressure and pulse were also normal.

#### Stool examination

The stool was processed for routine and microscopic examination and also concentration technique was performed. The eggs of *S. japonicum* were not present.

#### Blood tests

Patient refused the blood test on her follow up.

## Discussion

Many species of schistosomes have been reported from neighbouring countries of Nepal [7]. But only a few cases have been reported from Nepal to date since these parasites are uncommon in Nepal. Eggs with the appearance of *Schistosoma mansoni* have been reported in the inhabitants of Dhanusha district, Nepal, by Sherchand *et al*. and the seroprevalance for schistosomes of 18 % was found in the same district in 1996–1997, and 22.7 in the following year, i.e. 1998 in a follow-up study [8]. Devkota *et al*. confirmed the presence of *Schistosoma indicum* species by mitochondrial cytochrome oxidase I, 12S, 16S and 28S sequences (3675 bp) analysis of cercariae recovered from snails in Terai and hilly regions of Nepal from 2007 to 2014 [9]. But no cases of *S. japonicum* have been reported to date in Nepal – which might be due to the lack of the appropriate intermediate host, i.e. snail (*Oncomelania spps*) [8].

The first living case of *S. japonicum* in Malaysia has been reported by Murugasu *et al*. in 1973 in a patient with nephrotic syndrome. Leshem *et al*. diagnosed 7 out of 12 travellers with acute schistosomiasis caused by *S. japonicum* in 2009 in Laos [10]. The current case is also associated with a traveller having a history of recent arrival in Nepal with symptoms of acute schistosomiasis.

The source of infection is contact with fresh water contaminated with the cercariae and the mode of infection is skin penetration. The appropriate intermediate host, i.e. snail (*Oncomelania spps*) is required for the development of miracidium hatched from eggs excreted in the stool of definitive hosts like humans and other mammals [11]. The present case shows the symptoms of acute schistosomiasis and the stool examination also revealed the eggs of *S. japonicum*.

*S. japonicum* is susceptible to antihelminthic drugs and the drug of choice for acute schistosomiasis is Praziquantel (single dose) [4]. Due to the timely diagnosis and successful treatment of patient, the disease did not progress and other complications were avoided.

## Conclusion

The clinical condition due to the infestation of the parasite ranges from asymptomatic carrier to liver cirrhosis. The clinical symptoms and presentation of the disease are also similar to other helminthic infestation, hence only the careful examination of the stool, probably concentration technique along with the direct examination might increase the sensitivity of the detection of the egg of parasite, which help in appropriate treatment of the disease.

## References

[1] Carabin H, Johansen MV, Friedman JF, McGarvey ST, Madsen H (2011). Zoonotic schistosomosis (schistosomiasis). 2011;1(January). Available from.

[2] Katsurada F (1913). Schistosomiasis japonica. Zentralblatt fur Bakteriol Parasitenkunde, Infekt und Hyg Abt I. 1913;72(4/5):363–79. Available from: https://www.cabdirect.org/cabdirect/abstract/19142900427. https://www.cabdirect.org/cabdirect/abstract/19142900427.

[3] FEG C, Albert B, Max S (1930). Topley and Wilson’s Parasitology.

[4] Gillespie SH, Pearson RD (2001). Principles and Practice of Clinical Parasitology Principles and Practice of Clinical Parasitology.

[5] Zhou X-N, Guo J-G, Wu X-H, Jiang Q-W, Zheng J (2007). Epidemiology of schistosomiasis in the people's Republic of China, 2004. Emerg Infect Dis.

[6] Murugasu R, Wang F, Dissanaike AS (1978). Schistosoma japonicum-type infection in Malaysia--report of the first living case. Trans R Soc Trop Med Hyg.

[7] Agrawal MC (2012). Introduction BT - Schistosomes and Schistosomiasis in South Asia.

[8] Sherchand JB, Ohara H, Sherchand S, Matsuda H (1999). The suspected existence of Schistosoma mansoni in Dhanusha district, southern Nepal. Ann Trop Med Parasitol.

[9] Devkota R, Brant SV, Loker ES (2015). The Schistosoma indicum species group in Nepal: presence of a new lineage of schistosome and use of the Indoplanorbis exustus species complex of snail hosts. Int J Parasitol.

[10] Leshem E, Meltzer E, Marva E, Schwartz E (2009). Travel-related schistosomiasis acquired in Laos. Emerg Infect Dis.

[11] Drasar BS, Albert B, Max S (1932). Topley & Wilson’s Microbiology and Microbial infections.

